# Novel Calibration Strategy for Validation of Finite Element Thermal Analysis of Selective Laser Melting Process Using Bayesian Optimization

**DOI:** 10.3390/ma14174948

**Published:** 2021-08-30

**Authors:** Masahiro Kusano, Houichi Kitano, Makoto Watanabe

**Affiliations:** Research Center for Structural Materials, National Institute for Materials Science (NIMS), Sengen 1-2-1, Tsukuba, Ibaraki 305-0047, Japan; KITANO.Houichi@nims.go.jp (H.K.); WATANABE.Makoto@nims.go.jp (M.W.)

**Keywords:** selective laser melting, finite element thermal analysis, model calibration, thermographic image, bead on plate test, Bayesian optimization, nickel-based superalloy

## Abstract

Selective laser melting (SLM) produces a near-net-shaped product by scanning a concentrated high-power laser beam over a thin layer of metal powder to melt and solidify it. During the SLM process, the material temperature cyclically and sharply rises and falls. Thermal analyses using the finite element method help to understand such a complex thermal history to affect the microstructure, material properties, and performance. This paper proposes a novel calibration strategy for the heat source model to validate the thermal analysis. First, in-situ temperature measurement by high-speed thermography was conducted for the absorptivity calibration. Then, the accurate simulation error was defined by processing the cross-sectional bead shape images by the experimental observations and simulations. In order to minimize the error, the optimal shape parameters of the heat source model were efficiently found by using Bayesian optimization. Bayesian optimization allowed us to find the optimal parameters with an error of less than 4% within 50 iterations of the thermal simulations. It demonstrated that our novel calibration strategy with Bayesian optimization can be effective to improve the accuracy of predicting the temperature field during the SLM process and to save the computational costs for the heat source model optimization.

## 1. Introduction

Selective laser melting (SLM), which is classified into a powder bed fusion type in additive manufacturing methods, produces a near-net-shaped product by scanning a concentrated high-power laser beam on a thin layer of metal powder to melt and solidify it. The cyclic process of spreading a thin layer of powder and scanning the laser beam is repeated until the product is completed in the form designed by computer-aided design (CAD) software. Through the process, the material temperature periodically and rapidly rises and falls with melting and solidification with transformations. Such a thermal history affects the characteristic microstructure [1,2] and material properties [3,4], and it can also generate defects such as pores [5] and microcracks [6,7,8], and can cause distortion [9].

To understand the thermal history (temperature gradient, solidification rate, and cooling rate) in the SLM process, thermal analysis has been extensively performed using the finite element method (FEM) [10,11,12,13,14,15,16]. The FEM-based simulation solves the governing equations of heat transfer with boundary conditions, including a moving heat source model for laser scanning. Such heat source models have been originally studied for many years for welding processes, and some volumetric heat sources of various shapes and distributions have been proposed [10,14,17]. A double ellipsoidal power density model, proposed by Goldak et al. [17], can simulate shallow penetration for arc welding processes and deeper penetration for laser and electron beam processes by changing shape parameters. Accurate thermal analysis requires calibration of shape parameters, depending on material and process conditions. This model has been also applied to simulate the SLM process. The shape parameters have been calibrated so that the simulated bead shape (width, depth, and length) matches the one by the cross-sectional observation [10,11,12,13,14] or by in-situ monitoring with high-speed imaging [13].

In addition to the shape parameters, absorptivity in the heat source models, which means the heat efficiency of laser power in the process, depends on the material and process conditions. In the SLM process, the laser beam is reflected multiple times in the powder layer [18,19]. It also interacts with keyhole walls, a deep surface in the melting pool owing to the vapor recoil pressure of excessive laser energy [20]. Boley et al. [18] studied the absorptivity of a 1-µm-wavelength laser beam on a metal powder layer by ray-tracing calculations, and empirically measured the value by using direct calorimetric measurements. They proposed that the absorptivity of the powder layer can be predicted from that of the flat surface of its bulk metal. Trapp et al. [20] studied the effective absorptivity of a continuous 1070-nm-wavelength laser beam for some metal powders during melting. The in-situ direct calorimetric measurements revealed that the absorptivity considerably depended on laser power and scan speed (e.g., the absorptivity of 316 L stainless steel varied from 0.3 to 0.7 with the variation of laser power and scan speed). They have concluded that the measured absorptivities were different from both the one obtained by the powder layer measurements without melting and that of liquid metals estimated from the literature. Thus, the calibration of absorptivity is still necessary for the valid thermal analysis for the SLM process. However, to the best of our knowledge, no such thermal analysis using a heat source with calibrated absorptivity and shape parameters has been reported. This could be owing to the experimental difficulty of in-situ temperature measurements during the process.

Such thermal simulations are computationally expensive, and calibration is an iterative simulation process that uses different parameters to fit the simulation results with the experimental ones. Therefore, it is important to improve the efficiency of the calibration process. In short, the calibration process finds the parameters (explanatory variables *x*) to minimize the difference between experiment and simulation (an objective function f(*x*)). Bayesian optimization is known as a powerful tool to efficiently find minimum f(*x*) [21,22]. A Gaussian process model of f(*x*) is modified by a Bayesian update procedure at each new evaluation of f(*x*). One of the features of Bayesian optimization is the use of the acquisition function to determine the next point *x* to be evaluated. The acquisition function can balance between sampling at the points where the modeled objective function is low and exploring the areas that have not yet been modeled, resulting in efficient parameter optimization. For example, using Bayesian optimization, Kitano et al. [7,8] have successfully found an optimal laser irradiation conditions to avoid defects and cracks in SLMed nickel superalloys. Shiraiwa et al. [23] efficiently determined the heat source parameters in welding simulation using Bayesian optimization. In order to evaluate the accuracy of the calculation results and to improve it, the SLM process simulation also needs to be iterated many times to find the optimal heat source model parameters. Thus, Bayesian optimization could be an effective technique for the calibration of a heat source model for the SLM process simulation.

This paper proposes a novel calibration strategy for the heat source model of the FEM-based thermal analysis in the SLM process. The in-situ temperature measurements were conducted using high-speed thermography to calibrate the absorptivity. The cross-sectional bead shapes were quantified to calibrate the shape parameters of the heat source model. The shape parameters were explored using Bayesian optimization, which led to the smallest difference of the melt pool shape between the FEM calculations and the experimental measurements. The optimal parameters were compared with the ones of the grid search. These iterative calibration processes were performed via two-dimensional (2D) thermal simulations for computational efficiency. Three-dimensional (3D) thermal analysis with calibrated parameters was also performed and discussed in terms of bead shape, thermal history, and cooling rate.

## 2. Experiments

**Figure 1:**
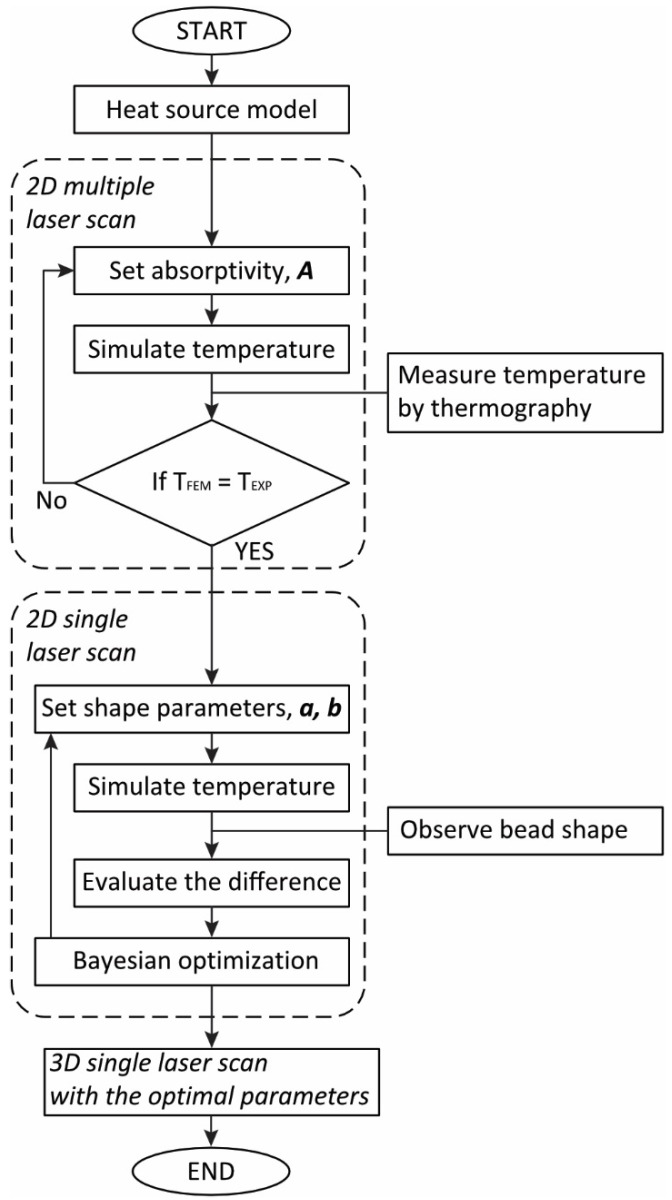
Flowchart of two-step of calibration strategy for the heat source model of FEM-based thermal analysis.

A two-step calibration strategy for the heat source model of FEM-based thermal analysis is shown as a flowchart in Figure 1. First, the absorptivity of the heat source model was calibrated to fit the temperature field by the simulation of multiple beads to the ones measured by thermography (Section 2.2). Second, Bayesian optimization offered better shape parameters for the heat source model, minimizing the difference between single bead simulation and cross-sectional observation results (Section 2.3). Thermal simulation models and Bayesian optimization are described in Section 3 and Section 4, respectively.

### 2.1. SLM Machine and Materials

**Figure 2:**
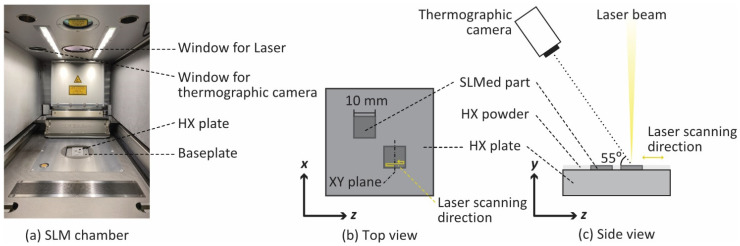
(**a**) A photo of the selective laser melting (SLM) chamber. Schematic images of in-situ temperature measurement of Hastelloy X (HX) by thermography: (**b**) top view and (**c**) side view.

Multiple and single laser scan tests were conducted using a commercial SLM machine, SLM 280 HL (SLM Solutions GmbH, Lübeck, Germany, Figure 2a). A thin layer of metal power on a base plate was scanned by a fiber laser beam with 1070-µm wavelength and 80-µm spot size in the chamber under an argon atmosphere. The laser power, scan velocity, and hatching space were set to 300 W, 1500 mm/s, and 100 µm, respectively. Nickel-based superalloy Hastelloy X (HX) powder (AMPERPRINT 0228 by Höganäs AB, Scania, Sweden) and 6-mm-thick baseplates (Alloy X by VDM Metals GmbH, Werdohl, Germany) were used in this study.

### 2.2. Thermographic Measurements

The temperature distribution due to multiple laser scans was measured by a high-speed thermography camera (FAST M350, Telops Inc., Quebec, Canada) through the silicon window of the chamber. The schematic image of the experimental setup is shown in Figure 2b,c. Two rectangular components with a dimension of 10 mm × 10 mm × 0.3 mm were prefabricated on the baseplate using SLM in advance. After that, when the top surface temperature was sufficiently lowered to ambient temperature, the platform with the substrate was lowered by 30 µm, and the additional powder layer was spread on the top surface. Then, the laser beam scanned in a zigzag pattern while the thermography camera measured the surface temperature.

The thermography camera has three filters to measure various temperature ranges: (1) 0–250 °C, (2) 106–350 °C, and (3) 265–780 °C. Thus, the measurements were performed for each filter. The sampling rate and exposure time of the camera were 345 Hz and 100 µs, respectively. In the preliminary experiment, the surface radiation of the SLMed HX part was separately calibrated by comparing the temperature measured by the camera with the one by a thermocouple in a furnace. The effects of the silicon window on the SLM machine were also calibrated in advance. Since the camera was mounted on the SLM machine at an angle of approximately 55°, the resolutions of images parallel and normal to the laser scan direction were 76 and 68 µm/pixel, respectively.

### 2.3. Bead on Plate Test

A single 10-mm-long bead was fabricated by scanning the laser beam on the powder layer (approximate 50 µm thick) on the baseplate. The single bead was cut perpendicular to the laser scanning direction, and the three cross sections were polished. Cross-sectional images of the backscattered electron composition (BEC) mode were taken via a scanning electron microscope (SEM: JSM-6010 LA, JEOL, Tokyo, Japan).

## 3. Thermal Analysis

The governing equation of heat transfer of an object with a density ρ, a specific capacity C, and a thermal conductivity k is as follows:

(1) $$\rho C\frac{\partial T}{\partial t}=\nabla \left(k\nabla T\right)+Q$$

where T, t, and Q are temperature, time, and heat source, respectively. The double ellipsoidal power density model [17] was applied to model the heat source by laser beam $q_{l}$, power P, and scan velocity v in the z-direction, as described as follows:

(2) $$q_{l}\left(x,\;y,\;z,\;t\right)=\{\begin{array}{c} \frac{6\sqrt{3}A_{1}P}{abc_{1}\pi \sqrt{\pi }}exp\left\{-3\left[{\left(\frac{x}{a}\right)}^{2}+{\left(\frac{y}{b}\right)}^{2}+{\left(\frac{z-vt}{c_{1}}\right)}^{2}\right]\right\}\;\;\;\;\;\;\;\;\left(z\geq vt\right)\\ \frac{6\sqrt{3}A_{2}P}{abc_{2}\pi \sqrt{\pi }}exp\left\{-3\left[{\left(\frac{x}{a}\right)}^{2}+{\left(\frac{y}{b}\right)}^{2}+{\left(\frac{z-vt}{c_{2}}\right)}^{2}\right]\right\}\;\;\;\;\;\;\left(z<vt\right) \end{array}$$

where $A_{1}$ and $A_{2}$ are the absorptivity forward and backward of the laser beam. The parameters a, b, $c_{1}$, and $c_{2}$ determine the shape of the volumetric heat source. In this study, it is assumed that $A_{1}=A_{2}=A$, $c_{1}=a$, and $c_{2}=2a$. These assumptions are common in the field of welding (e.g., reference [23]) and additive manufacturing (e.g., reference [16]).

**Figure 3:**
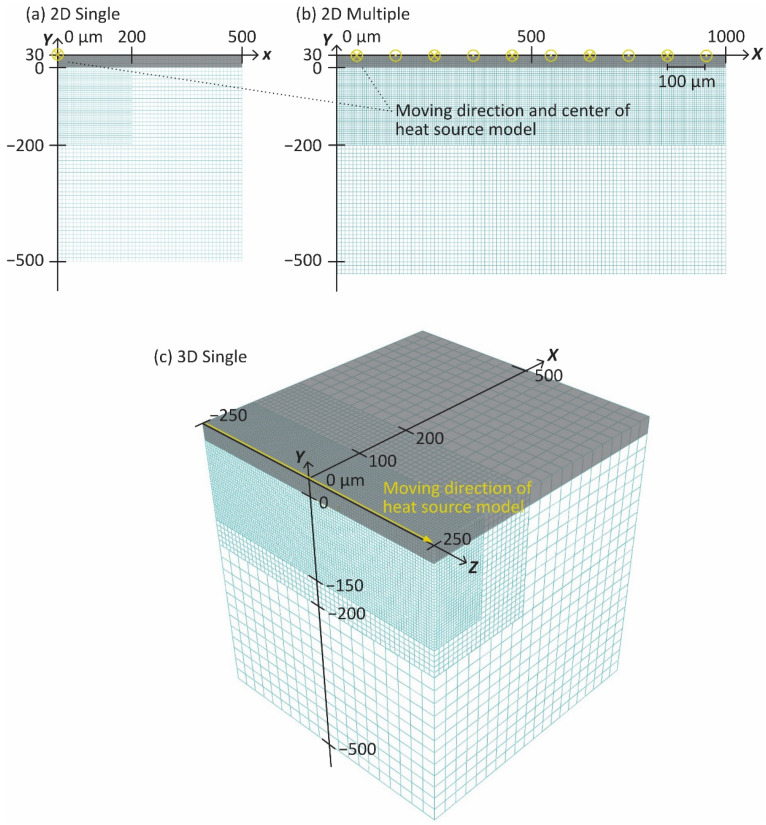
Thermal analysis models of (**a**) 2D single laser scan, (**b**) 2D multiple laser scan, and (**c**) 3D single laser scan.

Figure 3 shows the 2D models for the single and multiple laser scanning and the 3D model for the single laser scanning. The models consist of two components corresponding to a powder layer part of 30 µm thickness and a bulk part (gray and white elements in Figure 3, respectively). As shown in Figure 1, the absorptivity A was calibrated by comparing the temperature distributions measured by the thermography camera and simulated by the 2D multiple scan model (Figure 3b). The model of 1.0 mm × 6.3 mm corresponded to the center plane of the SLMed part and HX plate in Figure 2b. In order to realize the zig-zag pattern of the laser scan, the direction of the heat source model was alternately changed by 180° for every 10 mm of movement parallel to the Z-axis, and its center was shifted in the X direction by the same distance as the hatching space (100 µm). The simulations with different A were performed up to 0.5 s after the heat source had passed.

The 2D single scan model of 500 µm × 530 µm (Figure 3a) was prepared to optimize the shape parameters a and b by comparing it with the single bead on plate test. The heat source model with the calibrated parameters was also applied to the 3D single scan model of 500 µm × 530 µm × 500 µm (Figure 3c). These simulations were performed up to 0.75 ms after the heat source passed through the model. In order to reduce the computational cost, the Y-axis for the 2D single scan model (Figure 3a) and the YZ plane for 3D single scan model (Figure 3c) were used as the symmetry wall, and the center of the heat source model passed through the point (x, y)=(0, 30). The heat source model directions were indicated by symbols of ⊙ and ⊗ in Figure 3a,b, and by an arrow in Figure 3c. All boundaries except the top surface were assumed to be adiabatic. Heat loss due to natural convection and radiation, $q_{c}$ and $q_{r}$ on the top surface are defined as follows:

(3) $$q_{c}=h_{c}\left(T-T_{amb}\right)$$

(4) $$q_{r}=\sigma \varepsilon \left(T^{4}-T_{b}^{4}\right)$$

where $h_{c}$, ε, σ, and $T_{amb}$ are the convection coefficient, emissivity, Stefan-Boltzmann constant ($5.67\times 10^{-8}$ (W/m^2^·K^4^)), and ambient temperature (30 °C), respectively.

**Figure 4:**
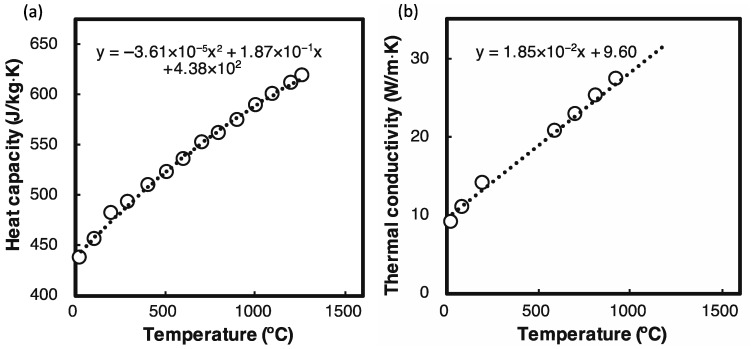
(**a**) Heat capacity and (**b**) thermal conductivity of HX (depending on temperature). Adapted from Ref. [24].

The material properties used in the simulation are listed in Table 1. As shown in Figure 4, for the bulk part of HX, $C_{solid}$ and $k_{solid}$ depended on temperature [24]. For the powder part, the density $\rho_{powder}$ and conductivity $k_{powder}$ were assumed to be $0.5\;\rho_{solid}$ and $0.05\;k_{solid}$, respectively. When the temperature exceeded the solidus temperature ($T_{s}$) of HX, the powder turned into the bulk by changing $k_{powder}$ to $k_{solid}$. Additionally, when the temperature exceeded the liquidus temperature ($T_{l}$), the conductivity $k_{liquid}$ was assumed to be 10 times larger than the one of the solid ($10\;k_{solid,\;\;1260\;{}^{\circ}C}=323$ W/(m·K)) to represent the heat transfer of the melting pool including the effect of fluidity. When the temperature was between $T_{s}$ and $T_{l}$, the properties of $k_{liquid}$ and $C_{liquid}$ were defined as follows [25,26]:

(5) $$\{\begin{array}{c} k=k_{solid}+\left(k_{liquid}-k_{solid}\right)\phi \\ C=C_{solid}+(C_{liquid}-C_{solid})\phi +L\frac{d\phi }{dT} \end{array}$$

where ϕ represents a phase fraction defined as $\left(T-T_{s}\right)/\left(T_{l}-T_{s}\right)$.

The thermal simulation was performed using the finite element code ABAQUS (ABAQUS/CAE 2019, Dassault Systems Simulia Corp., Johnston, RI, USA) with the user subroutines of the temperature and phase-dependent material properties and the moving volumetric heat source model. The 2D and 3D simulation used 4-node linear heat transfer quadrilateral and 8-node linear heat transfer brick elements (DC2D4 and DC3D8 in ABAQUS 2019), respectively. As shown in Figure 3, the mesh size was set to 5.0 µm for the part where large heat input was given by the moving heat source model, and larger sizes for other parts to save computational costs.

## 4. Bayesian Optimization

**Figure 5:**
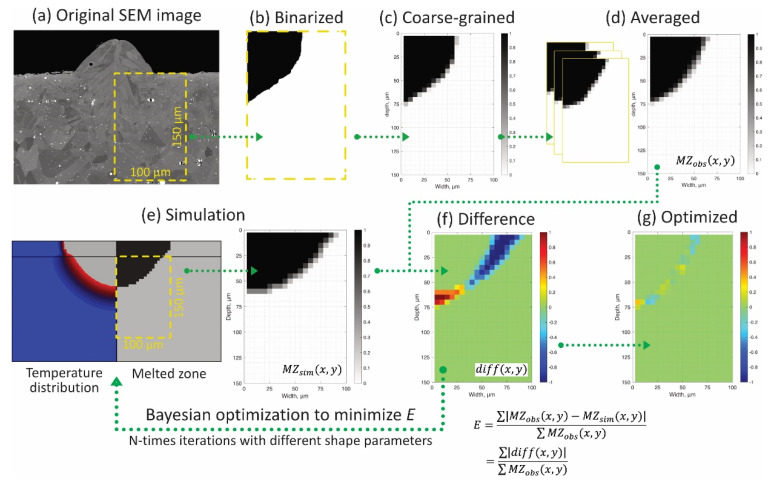
The procedure to evaluate the simulation error E. (**a**) Cross-sectional SEM image of the single bead with laser scanning, (**b**) binary image, and (**c**) coarse-grained image of the bead. (**d**) The visualization of the melted zone matrix $MZ_{obs}\left(x,y\right)$ averaged from three different cross sections. (**e**) The simulated melted zone $MZ_{sim}\left(x,y\right)$ (**right**) defined as the part exceeded $T_{s}$ during the thermal simulation (**left**). (**f**) Difference in bead shapes between the observation and simulation to evaluate the error E. (**g**) The optimized difference by iterative simulations with different parameters to minimize E with Bayesian optimization. The Black and white pixels with binary values correspond to the bead and baseplate, respectively.

The shape parameters a and b of the heat source model (Equation (2)) were calibrated by comparing the shape of beads by the observation (Section 2.3) and the thermal simulation (Section 3). Here, the error between the observation and the simulation was evaluated by the following procedure (see Figure 5). First, the area containing the right half of the melted zone in the SEM image (100 µm × 150 µm, surrounded by the yellow dashed rectangle in Figure 5a) was trimmed and manually binarized so that the grayscale of the pixels was set to 1 for the melted zone and 0 for the baseplate (Figure 5b). Second, as shown in Figure 5c, the binary image was divided into blocks to match the pixel size and simulation mesh size (coarse-graining). Several coarse-grained images were prepared for different cross sections and averaged into a single image (Figure 5d). This image is the visualized matrix of the melted zone $MZ_{obs}\left(x,y\right)$, and its components range is from 0 to 1. Here, x and y are the coordinates in the width and depth directions of the melted zone, respectively. In the finite element simulation, the melted zone was defined as the part whose temperature exceeded $T_{s}$ during laser scanning. As shown in Figure 5e, the melted zone was also outputted as binary values for each integral point of the elements in the corresponding area to the SEM image (100 µm × 150 µm), and the average values of four integral points in the elements were used to obtain the matrix of the melted zone $MZ_{sim}\left(x,y\right)$. Subtracting the simulated matrix $MZ_{sim}\left(x,y\right)$ (Figure 5e) from the observed matrix $MZ_{obs}\left(x,y\right)$ (Figure 5d) yields a different matrix (diff(x,y)) or an image, as shown in Figure 5f. Warm colors indicate that the simulated melt was smaller than the observed bead shape, and cold colors mean the opposite. Finally, the simulation error E was defined as follows:

(6) $$E=\frac{\sum \left|MZ_{obs}\left(x,y\right)-MZ_{sim}\left(x,y\right)\right|}{\sum MZ_{obs}\left(x,y\right)}=\frac{\sum \left|diff\left(x,y\right)\right|}{\sum MZ_{obs}\left(x,y\right)}$$

The optimal shape parameters to minimize E were found by Bayesian optimization by using Statistics and Machine Learning Toolbox in MATLAB (R2020b, MathWorks, Natick, MA, USA). Bayesian optimization uses a Gaussian process model to find the point x that minimizes the objective function y=f(x). The Gaussian process model of f(x) is modified by Bayesian updates each time a new point is acquired. The Gaussian process is defined as mean μ(x;θ) and covariance kernel function $k\left(x,x^{\prime };\theta \right)$. Here, θ is a vector of kernel parameters. The kernel function $k\left(x,x^{\prime };\theta \right)$ can significantly affect the quality of a Gaussian process regression, and the Bayesian optimization in the MATLAB toolbox uses the automatic relevance determination (ARD) Matérn 5/2 kernel, $K_{M52}\left(x,\;x^{\prime }\right)$ [22]:

(7) $$K_{M52}\left(x,\;x^{\prime }\right)=\theta_{0}\left(1+\sqrt{5}r+\frac{5}{3}r^{2}\right)exp\left(-\sqrt{5}r\right)$$

(8) $$r^{2}=\overset{D}{\underset{d=1}{\sum }}\frac{\left(x_{d}-{x^{\prime }}_{d}\right)}{\theta_{d}^{2}}$$

where hyperparameters $\theta_{0}$ and $\theta_{1:D}$ are the covariance amplitude and the length scales, respectively.

The next evaluation point is determined by the acquisition function a(x) that evaluates the “goodness” of the point x based on the posterior distribution $Q(f|x_{i},y_{i}\;for\;i=1,\ldots ,t)$ of the Gaussian process model. The acquisition function can balance sampling points around the low objective function (exploitation) and searching for the regions that have not yet been fully explored (exploration). Thus, the efficiency of parameter retrieval by Bayesian optimization depends on the acquisition functions. In this study, three acquisition functions were examined: (1) expected improvement (EI), (2) probability of improvement (PI), and (3) lower confidence bound (LCB) [21,22,27]. The EI acquisition function evaluates the expected amount of improvement in the objective function, ignoring values that increase the objective function. EI is defined as follows:

(9) $$\;\mathrm{EI}=E\left[max\left(0,\mu_{Q}\left(x_{best}\right)\right)-f\left(x\right)\right]$$

where E(x) indicates the expected value of x, $x_{best}$ is the lowest posterior mean point, and $\mu_{Q}\left(x_{best}\right)$ is the lowest value of the posterior mean. The PI acquisition function calculates the probability that the new point x leads to a better objective function value, modified by the margin parameter m which takes the estimated noise standard deviation of the Gaussian process model:

(10) $$\mathrm{PI}=\mathrm{CDF}\left(\frac{\mu_{Q}\left(x_{best}\right)-m-\mu_{Q}\left(x\right)}{\sigma_{Q}\left(x\right)}\right)$$

where CDF indicates the cumulative distribution function of the standard normal distribution, and $\sigma_{Q}\left(x\right)$ is the standard deviation. The LCB function minimizes the curve of $2\sigma_{Q}\left(x\right)$ at each point:

(11) $$\mathrm{LCB}=2\sigma_{Q}\left(x\right)-\mu_{Q}\left(x\right)$$

In this study, the search point x corresponds to the heat source shape parameters a and b, and the objective function y=f(x) is defined as the difference in bead shapes between simulations and experiments. For further details, see the literature [21,22,27]. The search areas of a and b were (10, 100 µm) and (50, 200 µm), respectively. The single bead simulation and newly selected parameters were iterated 50 times. A grid search was also performed for the same area of shape parameters for comparison. The grid sizes of both parameters a and b were 5.0 µm.

## 5. Results

### 5.1. Absorptivity Calibration

**Figure 6:**
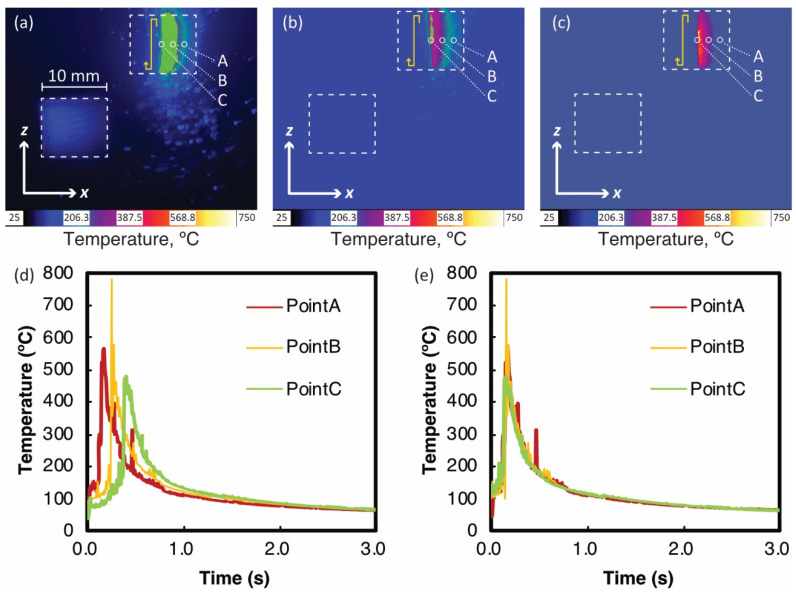
Temperature distributions on the top surface measured by thermography during multiple laser scanning: (**a**) 0–250 °C, (**b**) 106–350 °C, (**c**) 265–780 °C, (**d**) temperature histories at points A, B, and C, and (**e**) temperature histories at points B and C are shifted by a time lag to match the temperature history at point A.

Figure 6a–c show the temperature distributions during multiple laser scans measured by the thermography camera with different filters. These images show the temperature distribution when the laser beam scans the middle of the sample. Point C in the figures indicates the sample center point. Points A and B are 1.6 and 3.2 mm away in the X-direction from point C, respectively. As shown in Figure 6d, the temperature histories with different filters were integrated into one for each point. The laser beam scanned through the sample surface (10 mm × 10 mm) at the speed of 1500 mm/s and the hatching space of 100 µm, so there was a time lag of approximately 0.1 s between the three curves. When the peak positions are aligned as shown in Figure 6e, the three curves are in good agreement, indicating that the thermal histories at these points are almost the same.

The actual temperature at the laser-irradiated spots should exceed $T_{s}$ and $T_{l}$. Therefore, another filter for the high-temperature range of 750–2500 °C was also examined. However, such high temperatures could not be measured by the thermography camera. This can be owing to the temporal and spatial resolution limitations of the camera. Zhirnov et al. [28] used an advanced thermography camera with a 20,000 frame rate and 3.07 µm/pixel resolution to measure sharp laser spots over 1900 °C. However, as discussed later, the temperature variation during cooling period below 500 °C was sufficient to calibrate the absorptivity.

**Figure 7:**
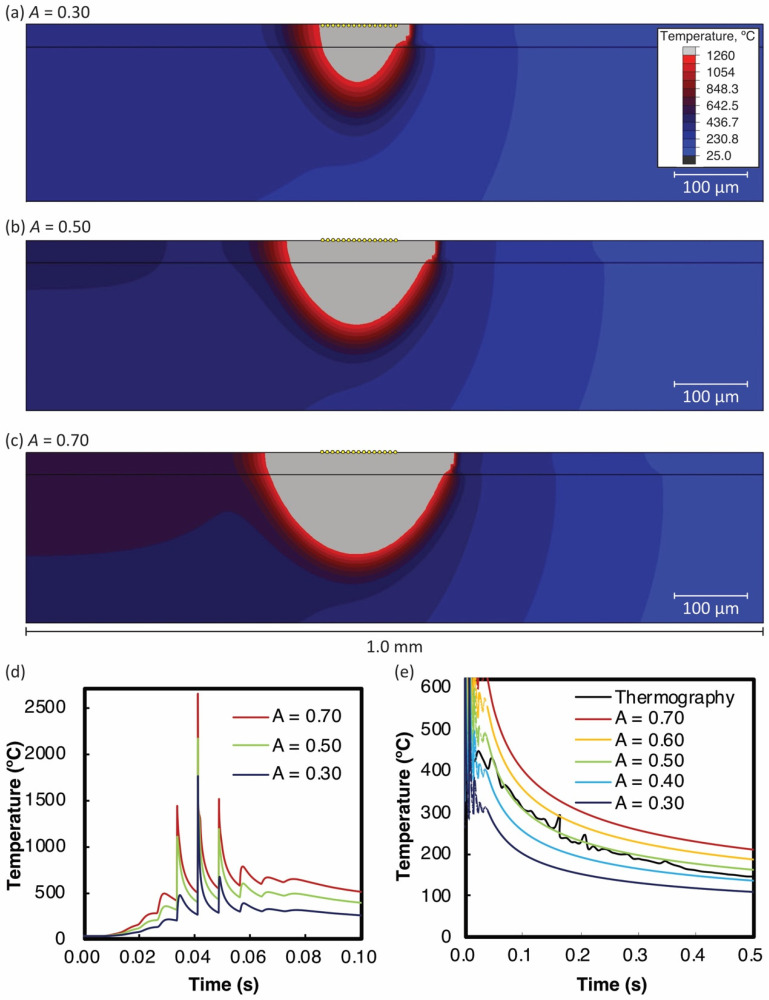
Temperature distributions by thermal simulation of multiple laser scanning with different absorptivities A: (**a**) 0.30, (**b**) 0.50, (**c**) 0.70, (**d**) cyclic thermal histories, and (**e**) cooling process from maximum temperature. In (**a**–**c**), the average temperature in the top center of 14 nodes is indicated in yellow. Black lines in (**e**) represent temperatures measured by the thermography camera (Figure 6e).

Figure 7a–c show the temperature distribution by the 2D thermal simulation of multiple laser scans with A= 0.30, 0.50, and 0.70 when the heat source is in the center of the model. Here, it was assumed that the shape parameters a and b were 50 and 125 µm, respectively. The part whose temperature exceeds $T_{s}$ is shown in gray indicating the melting pool. It was evident that the size of the melting pool increased as the absorptivity increased. The thermography camera has a spatial resolution of 68 µm, so we averaged the temperatures of the 14 nodes in the top center (yellow points in Figure 7a–c). Figure 7d,e show the average thermal histories on various axis scales. Figure 7d shows the cyclic temperature rise and fall owing to adjacent laser paths. Such heating by adjacent paths was also recognized from the asymmetry of the melting pool in Figure 7a–c.

Figure 7e shows the cooling process from the maximum temperatures for different absorptivity assumptions. The black line in the figure indicates the experimental data measured by the thermography camera. The results indicate that “0.50” seems suitable for the absorptivity A to reproduce the surface temperature variation in the thermal analysis. As described in Section 1, the absorptivity of the heat source model includes the effects of the powder layer and the surface shape of the melting pool. There is no data available for the absorptivity of HX. For the case of 316L stainless steel powder, its absorptivity was reported as in the range from 0.3 to 0.7 for the different laser energy densities [20]. The estimated value, A ~0.50 for HX in the present study was in a similar range and was considered appropriate.

### 5.2. Shape Parameter Calibration of Heat Source Model

**Figure 8:**
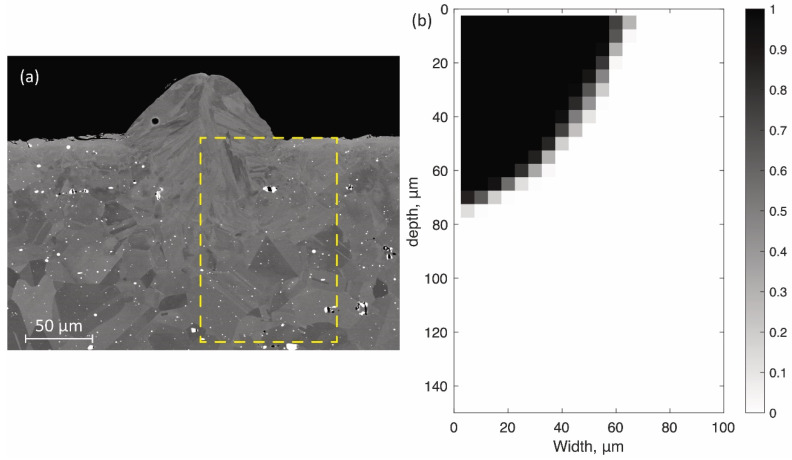
(**a**) Cross-sectional SEM image of the single bead with laser scanning. A yellow rectangle indicates the area to be processed. (**b**) The visualization of the melted zone matrix $MZ_{obs}\left(x,y\right)$. Black and white pixels with binary values correspond to the bead and baseplate, respectively.

Figure 8a shows one of cross-sectional SEM images of the single bead using the SLM process at the power of 300 W and the speed of 1500 mm/s. The mean width and depth of melted zone at three different cross sections (see Figure 8a) were 60.8 ± 4.79 µm and 69.8 ± 1.22 µm, respectively. Figure 8b shows $MZ_{obs}\left(x,y\right)$ processed by the procedure described in Section 4. The gray pixels in the averaged image in Figure 8b were just observed along the boundary between the melted and non-melted zones. This indicates that the shapes of the melted zone at the three different cross sections were almost identical.

**Figure 9:**
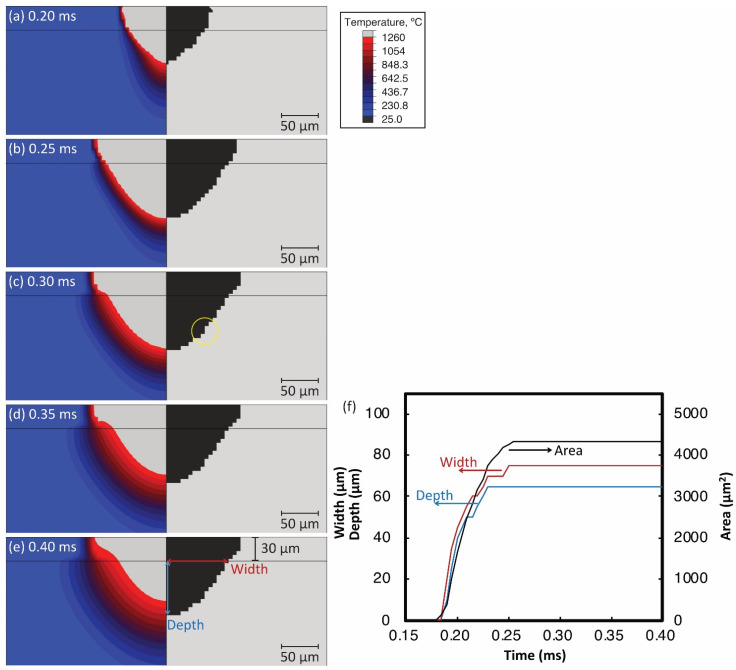
Temperature distribution (**left**) and melted zone (**right**) simulated by 2D thermal analysis with parameters a= 25 µm, b= 125 µm, and A= 0.50 at the moment of (**a**) t= 0.20 ms, (**b**) 0.25 ms, (**c**) 0.30 ms, (**d**), 0.35 ms, and (**e**) 0.40 ms. (**f**) The width, depth, and area of the melted zone with the time.

Figure 9a–e show the change of temperature distribution and the formation of melted zone during the 2D thermal simulation of a single laser scan with A= 0.50, the shape parameters a= 50 µm, and b= 125 µm. Figure 9f shows the simulated width, depth, and area of the melted zone over time. In this simulation, the center of volumetric heat source passed through the XY plane at 0.25 ms so that the melting pool and melted zone became larger from 0.185 to 0.25 ms (see Figure 9a,b,f). After the heat source passed through, the melted zone became slightly wider at 0.255 ms (see a yellow circle in Figure 9c). Then, the melting pool gradually got small as the inputted heat was transferred to the surrounding area (Figure 9d,e).

**Figure 10:**
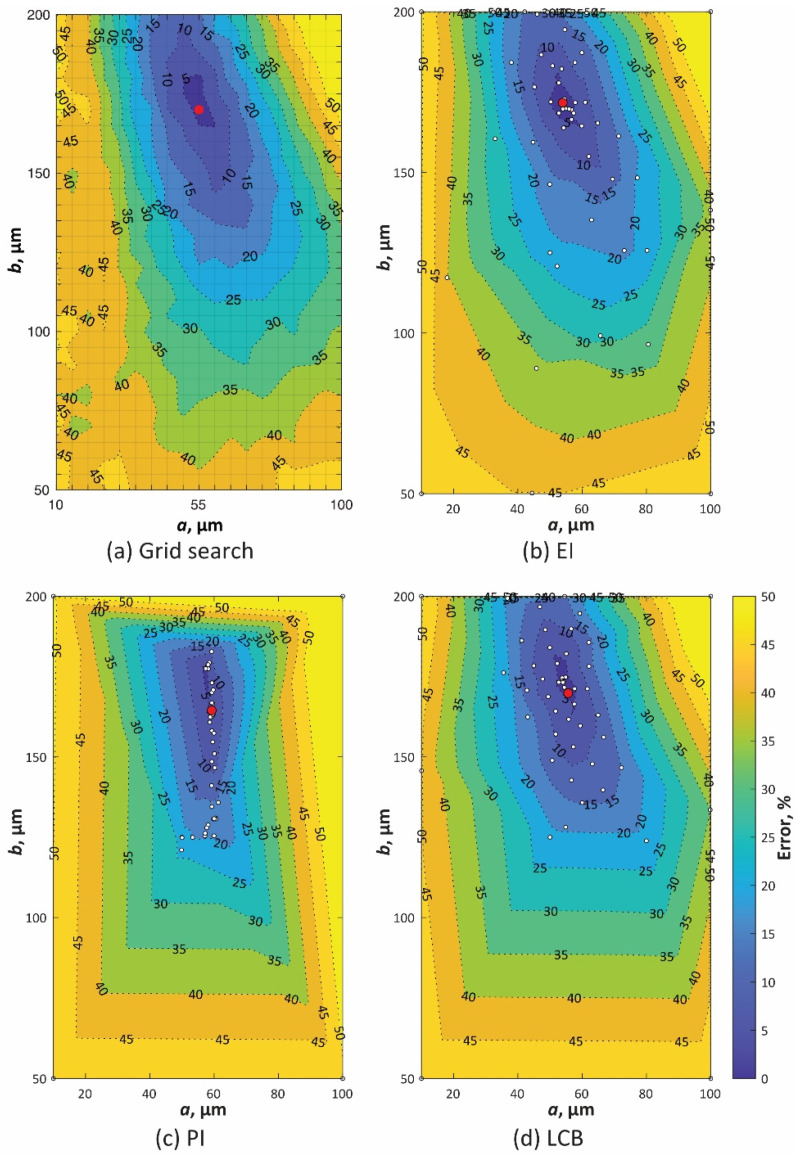
Contour map of error E simulated by (**a**) grid search and Bayesian optimization with the acquisition function of (**b**) EI, (**c**) PI, and (**d**) LCB. The white circles represent the searched points, and the red circles are optimal parameters to minimize E.

**Figure 11:**
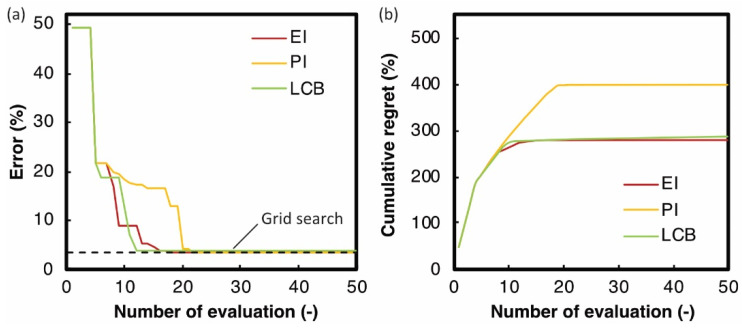
(**a**) Error E and (**b**) cumulative regret R with the number of evaluations.

**Figure 12:**
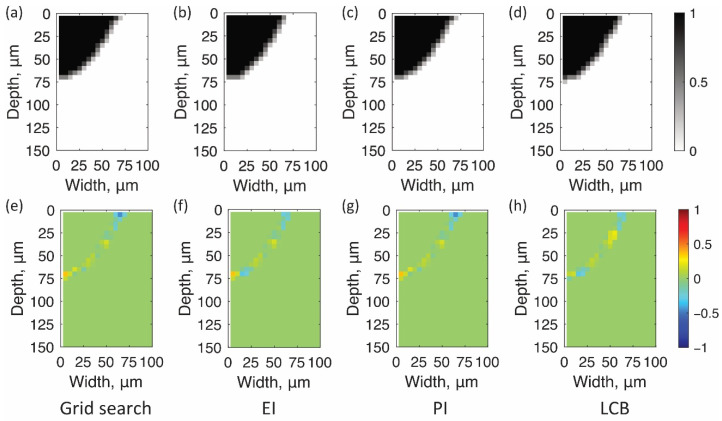
Bead shapes by simulation with optimal parameters (**a**–**d**) and their differences from the observation (**e**–**g**). (**a**) and (**e**) are the grid search and Bayesian optimization with the acquisition function of (**b**) and (**f**) EI, (**c**) and (**g**) PI, and (**d**) and (**h**) LCB.

According to the procedure described in Section 4, the optimal shape parameters a and b to minimize error E were searched by Bayesian optimization. Figure 10b–d show contour maps of the errors searched by various acquisition functions. White circles indicate the searched parameters. Figure 11a shows the minimum errors through the search process with the number of evaluations. Figure 10a also shows the contour map of the errors by the grid search with more than 600 times simulations. The search parameters are on the grid lines in the figure. All these contour maps show that the error has one local minimum point around (55 µm, 170 µm). Instead of reducing the search for larger error areas, Bayesian optimization with all acquisition functions better exploited the objective functions around the minimum point. As a result, the optimal parameters with errors equivalent to those of grid search were achieved within 25 iterations by Bayesian optimization (Figure 11a). The optimal parameters for the grid search and each acquisition function are shown in red circles in Figure 10 and are listed in Table 2. Three acquisition functions represented similar shape parameters for minimizing E. Figure 12 shows the bead shape by simulation $BS_{sim}\left(x,y\right)$ with the optimal parameters and its difference matrix diff(x,y). As shown in these figures, all the Bayesian optimizations with the three acquisition functions succeeded in efficiently detecting the shape parameters that fit the shape of the beads.

The search efficiency of each acquisition function was evaluated by cumulative regret R, defined as follows [18,20]:

(12) $$R=\underset{i}{\sum }\left(E_{i},-E_{min}\right)$$

where $E_{i}$ is the ith error and $E_{min}$ is the minimum error. The smaller the cumulative regret, the more efficient search in the optimization. As shown in Figure 11b, LCB and EI efficiently searched for the best parameters than PI because of the balance of exploitation and exploration. In any case, compared to the grid search, which required more than 600 calculations, Bayesian optimization with any acquisition function was much more efficient in determining optimal shape parameters.

### 5.3. 3D Thermal Analysis with Calibrated Parameters

The novel two-step calibration proposed above was performed using a 2D FEM-based thermal simulation model that takes computational costs into account. 3D thermal analysis requires enormous computational time and resources, as the number of meshes significantly increases with the length of the model in the Z-direction. Thus, it is not feasible to use such a 3D model for the calibration procedure with iterative simulations even though Bayesian optimization can significantly reduce the number of iterations. However, a 3D thermal analysis can be desirable to understand the temperature distribution better and combine the results of thermal simulation with other simulations (such as thermal stress analysis [9], thermal elastoplastic analysis [7,8], and grain growth simulation [2]). Consequently, a rational process is to use the 2D model to calibrate the parameters and use the calibrated parameters to simulate the 3D model. Here, the process is validated to compare 3D and 2D models with parameters calibrated in terms of bead shape, thermal history, and cooling rate.

**Figure 13:**
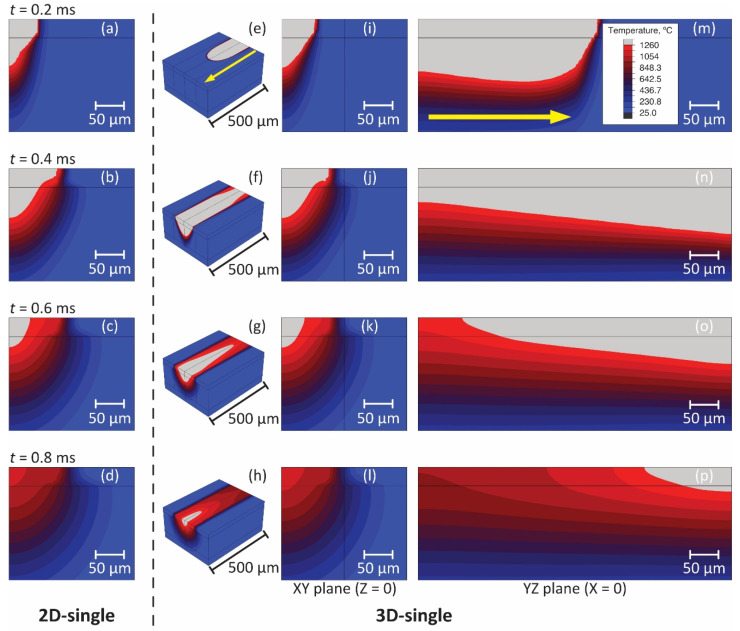
Temperature distributions simulated using the 2D single (**a**–**d**) and 3D single (**e**–**p**) models with calibrated parameters (A,a,b)=(0.5, 53.95, 171.67) at different time steps t= 0.2, 0.4, 0.6, and 0.8 ms. (**i**), (**j**), (**k**), and (**l**) are XY planes at Z= 0.0 µm, and (**m**), (**n**), (**o**), and (**p**) are YZ planes at X= 0.0 µm. Yellow arrows indicate a laser scanning direction.

**Figure 14:**
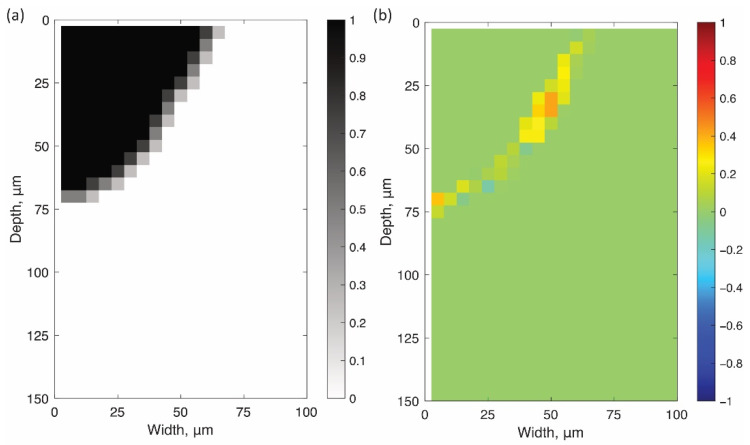
(**a**) Melted zone $MZ_{sim}\left(x,y\right)$ at Z=0 µm in the 3D single model, and (**b**) its difference diff(x,y) from the observation (Figure 8b).

Figure 13 shows the 2D and 3D temperature distributions of the single laser scan with the calibrated absorptivity and the shape parameters [(A,a,b)=(0.5, 54.0, 171.7)]. Figure 13a–d are the results of the 2D calculations and Figure 13e–p are the ones by the 3D analysis at the different times and for the different viewing angles. The 3D temperature distribution in Figure 13e–g were shown as a mirror reflection, using the symmetry in the YZ plane. Comparing Figure 13i–l and Figure 13a–d, the temperature distribution at the XY plane in the 3D model was almost the same as that in the 2D model. Similar to the calibration procedure in Section 4, $MZ_{sim}\left(x,y\right)$ and diff(x,y) were evaluated at the XY plane in the 3D model. It is clear from Figure 14 that the melted zone simulated in the 3D model was consistent with the bead shape on the cross section. The error E of the 3D simulation was 4.73% and the difference was only 1.25% comparing to the 2D simulation. In most cases, the error was small enough to justify the 3D simulation with the shape parameters optimized in the 2D model.

**Figure 15:**
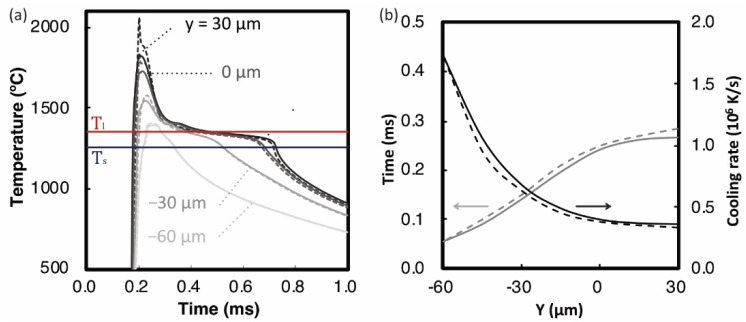
(**a**) Simulated thermal history at Y= 30, 0, −30, and −60 µm and (X,Z)= (0.0, 0.0) of the 3D model and (**b**) solidification time from $T_{l}$ to $T_{s}$ and the cooling rate in Y direction. Solid and broken lines indicate the results of single 3D and 2D models, respectively.

Figure 15a shows thermal histories at Y= 30, 0, −30, and −60 µm and at (X,Z)= (0.0, 0.0) in the 3D model. For comparison, the dashed lines in the figure represent the thermal histories of the 2D model at the same coordinate points. The thermal histories of both models showed almost the same behavior. Red and blue lines in Figure 15a represent the temperatures of $T_{l}$ and $T_{s}$. The cooling time from $T_{l}$ to $T_{s}$ plotted in Figure 15b increased in the Y direction (as the depth of melting pool decreased). This indicates that shallower the melting pool, the more slowly it solidifies. The temperature difference between $T_{l}$ to $T_{s}$ divided by the time corresponds to an average cooling rate during the phase transformation from liquid to solid. The cooling rate decreased from 1.7 × 10^6^ to 3.6 × 10^5^ K/s in the Y direction. Again, the 3D and 2D models showed the same trends for the cooling rate.

## 6. Discussion

This section discusses the novel calibration strategy for the thermal analysis in the SLM process in terms of the computational efficiency by 2D model and Bayesian optimization, the similarity between the 2D and 3D models, the validity and limitation of the calibrated heat source model, and the advantage of the FEM-based thermal analyses over more advanced simulations for the SLM process.

In this study, the shape parameters of the heat source model were calibrated using the 2D thermal simulation models, and it was confirmed that the calibrated parameters were also valid for the 3D thermal simulation. The 2D single scan model takes less than 2 min on a desktop personal computer (PC) with an Intel Core i7 CPU (Intel Corporation, Santa Clara, CA, USA), while the 3D model takes about 2 h. Thus, as well as Bayesian optimization, the iterative simulations with the 2D single scan model could find the optimal shape parameters within 2 h (50 iterative simulations). The 3D single laser scan simulation with the calibrated parameters also showed an error of 4.73% compared to the cross-sectional observations. Thus, the novel calibration strategy effectively reduces the computational cost.

As confirmed in Section 5.3, the 2D and 3D single laser scan simulations showed almost identical melted zone, thermal history, and cooling rate. This implies that the heat input by the heat source model was mainly transferred in the X and Y directions (perpendicular to the moving direction of the heat source) rather than in the Z direction in the 3D model. Note that these simulation models were for the middle part of a sufficiently long single laser scan. At the ends of single laser scan, the cooling rate in the 2D model will be slower than in the 3D model and the experiment because the actual input heat transfers to the surrounding substrate in three dimensions.

The parameters of the double ellipsoidal power density model were calibrated using both the temperature measured by the thermography camera and the cross-sectional bead shape observed by SEM. In this study, as a first step, the parameters $c_{1}$ and $c_{2}$ in Equation (2) were assumed to be a function of a as in reference [16,23]. Some researchers reported the calibration of the shape parameters by observing the top surface of the melting pool during a laser scan using high-speed imaging [13]. Thus, in-situ observation of such melting pools helps make thermal simulations more effective. However, studying the shape of the top bead by FEM-based thermal analysis is not practical. The surface tension of the melting pool makes the shape of the top bead semi-circular. However, since the current FEM-based thermal analysis does not take such surface tension into account, the shape of the top bead becomes unrealistic (see Figure 9). Thus, if the size of the melting pool observed by high-speed imaging is used for calibration, the effects of surface tension must be considered by using computational fluid dynamics (CFD).

Absorptivity and bead shape parameters depend on laser scan conditions. As described in the Introduction section, the absorptivity is significantly dependent on the laser power and scanning speed [20]. The authors also studied the shape of a single bead of HX, which can be deep or shallow depending on the laser power and speed of the SLM process [7]. Thus, parameters calibrated for the specific laser scan conditions may not be useful for other conditions. One solution is to define the parameters as a function of laser scan conditions and calibrate the coefficients of the function by experimental measurements with various conditions. Suppose such a heat source model is developed as a function of laser scan conditions. In that case, the thermal simulation applicable to any laser scan conditions will be significantly useful for other simulations related to SLM processes such as prediction of microstructure evolution and microcracking.

One might be concerned about the effects of shape parameters on the multiple laser scan simulation for absorptivity calibration. In Section 5.1, the shape parameters a and b were assumed to be 50 and 125 µm, respectively. When the same simulations with some different values of shape parameters within the search area, the surface temperature differed by only 10 °C at most in the time range for the calibration (from 0 to 0.5 s after laser scanning). This value was sufficiently small compared to the temperature change (more than 50 °C, see Figure 7e) when the absorptivity was varied by 0.1. Thus, the two-step calibration strategy is reasonable if the first decimal place of absorptivity A is to be determined.

Fundamentally, such a heat source model with some fitting parameters is necessary because the current FEM-based thermal simulation does not directly model laser absorption and reflection in the powder layer and the fluidity of the melting pool. This can be a drawback compared with more advanced simulations such as ray-tracing simulation of laser beams on powder particles and thermal analysis with CFD. However, FEM-based thermal simulations with such a simplified heat source model can use less computational costs and resources. Again, the 3D FEM-based thermal simulation takes approximately 2 h to complete a single bead simulation on the desktop PC. On the other hand, the CFD-based thermal simulations of comparable scale take even longer, and need to be parallelized. Thus, FEM-based thermal analysis helps to efficiently understand the large-scale thermal behavior of multiple laser scans and multiple layers.

## 7. Conclusions

This study proposed a novel calibration strategy of the heat source model for FEM-based thermal analysis of the SLM process. Empirical data from in-situ temperature measurements by high-speed thermography and SEM observation of single bead cross sections were used to calibrate the heat source model absorptivity and shape parameters. The findings obtained in this study were as follows:

- Bayesian optimization allowed to efficiently find the optimal parameters with an error of less than 4% within 50 iterations of the thermal simulations.
- The use of the 2D models for the parameter calibrations significantly reduced the cost of iterative simulations.
- The 3D single laser scan simulation with the calibrated parameters showed almost the same results of the 2D simulation in thermal history, melted zone, and cooling rate.

Thus, our novel calibration strategy for thermal analysis is feasible to precisely and efficiently understand the thermal history of the actual SLM process.

## Figures and Tables

**Table 1:** Material properties of Hastelloy X (HX) used for thermal analysis. Adapted from Ref. [24].

Material Properties	Value
Density for solid, *ρ_solid_*	8240 kg/m^3^
Density for powder, *ρ_powder_*	4120 kg/m^3^
Heat capacity for liquid, *C_liquid_*	674 J/kg K
Thermal conductivity for liquid, *k_liquid_*	323 W/m K
Latent heat, *L*	276 kJ/kg
Solidus temperature, *T_s_*	1260 °C
Liquidus temperature, *T_l_*	1355 °C
Heat transfer coefficient, *h_c_*	10 W/m^2^ K
Emissivity, *ε*	0.3

**Table 2:** Optimal parameters found by grid search and Bayesian optimization with different acquisition functions and their errors.

Search Strategy	Acquisition Function	*a*, µm	*b*, µm	Error, %
Bayesian optimization	EI	54.0	171.7	3.48
	PI	59.3	164.5	3.48
	LCB	55.7	169.8	3.68
Grid search	-	55.0	170.0	3.48

## Data Availability

Data sharing not applicable.
